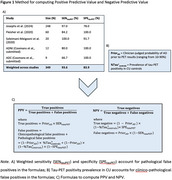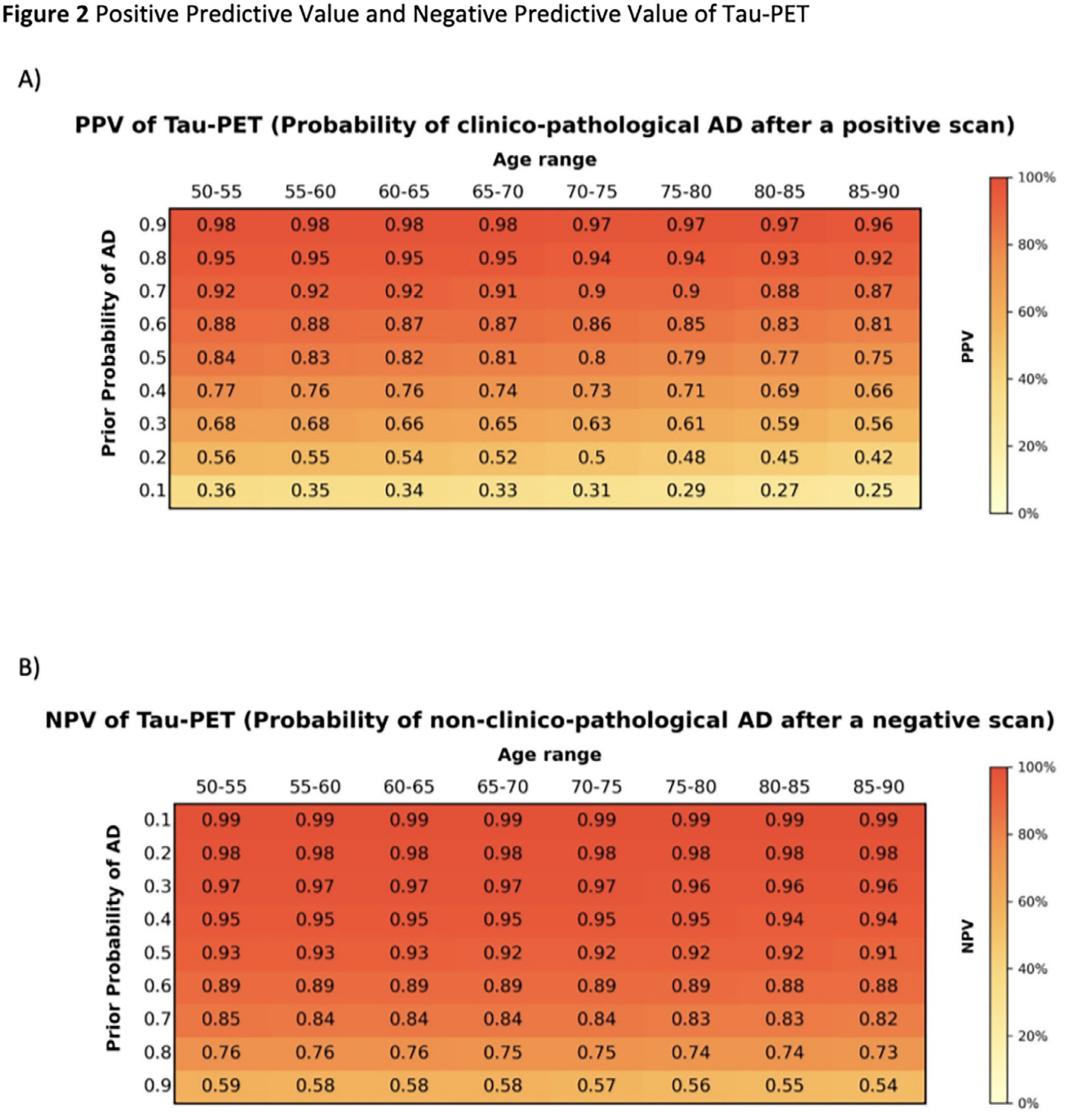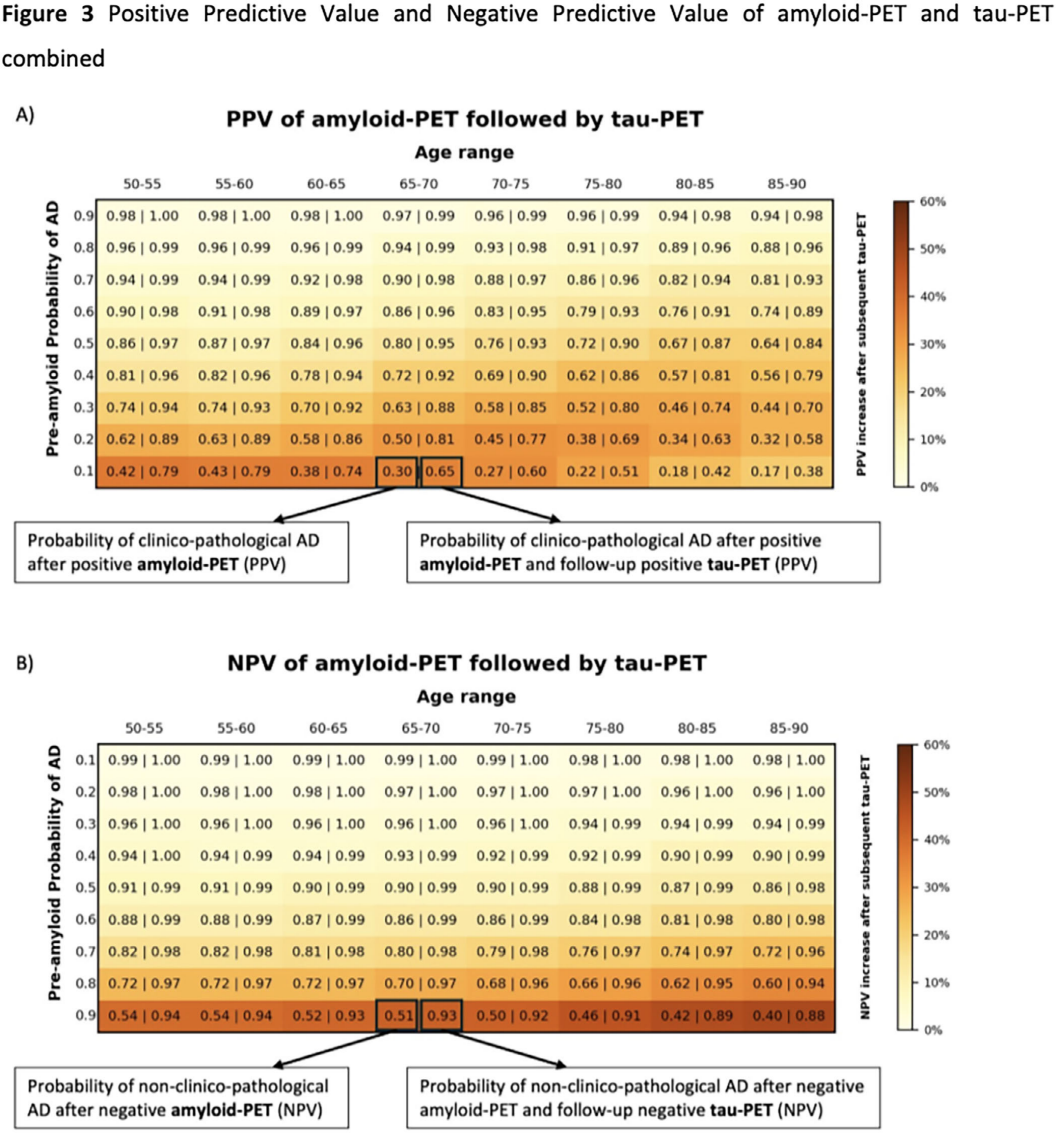# A probabilistic framework for the diagnostic utility of tau‐PET

**DOI:** 10.1002/alz70862_110823

**Published:** 2025-12-23

**Authors:** Bastiaan G J van Tol, Colin Groot, Elsmarieke van de Giessen, Yolande A.L. Pijnenburg, Emma M. Coomans, Rik Ossenkoppele

**Affiliations:** ^1^ Alzheimer Center Amsterdam, Neurology, Vrije Universiteit Amsterdam, Amsterdam UMC location VUmc, Amsterdam, Noord‐Holland Netherlands; ^2^ Amsterdam Neuroscience, Neurodegeneration, Amsterdam, The Netherlands, Amsterdam, Noord‐Holland Netherlands; ^3^ Alzheimer Center Amsterdam, Neurology, Vrije Universiteit Amsterdam, Amsterdam UMC location VUmc, Amsterdam Netherlands; ^4^ Amsterdam Neuroscience, Neurodegeneration, Amsterdam Netherlands; ^5^ Department of Radiology and Nuclear Medicine, Amsterdam UMC, Vrije Universiteit Amsterdam, Amsterdam Neuroscience, Amsterdam Netherlands; ^6^ Amsterdam Neuroscience, Brain Imaging, Amsterdam Netherlands; ^7^ Alzheimer Center Amsterdam, Department of Neurology, Amsterdam UMC, location VUmc, Amsterdam Netherlands; ^8^ Department of Neurology, Alzheimer Center Amsterdam, Amsterdam Neuroscience, Vrije Universiteit Amsterdam, Amsterdam Netherlands; ^9^ Clinical Memory Research Unit, Department of Clinical Sciences, Lund University, Lund Sweden

## Abstract

**Background:**

The tau‐PET radiotracer [18F]flortaucipir enables in vivo detection of tau pathology in Alzheimer’s Disease (AD) and has recently been FDA‐ and EMA‐approved for clinical use. To support its implementation, we assessed tau‐PET's positive predictive value (PPV) and negative predictive value (NPV) for clinico‐pathological AD while accounting for age, amyloid‐status, and pre‐PET diagnostic certainty.

**Method:**

We computed the PPV and NPV of tau‐PET using a formula that considers two types of false‐positivity: clinico‐pathological (positive tau‐PET, yet tau does not significantly contribute to cognitive decline) and pathological (positive tau‐PET, yet no/low tau is found at autopsy; Figure‐1C). A systematic review yielded a weighted sensitivity of 93.6% and specificity of 83.9% for [18F]flortaucipir to detect postmortem Braak V/VI tau pathology (*N* = 349; Figure‐1A). PPV and NPV were calculated across age groups using previously derived tau‐PET positivity prevalence estimates in cognitively unimpaired individuals and hypothetical clinician‐estimated prior probabilities of clinico‐pathological AD (Figure‐1B), resulting in the probability that cognitive impairment is primarily caused by AD‐related tau pathology. We evaluated the PPV and NPV of tau‐PET as a standalone biomarker and in combination with amyloid‐PET.

**Result:**

The PPV of standalone tau‐PET was highest in individuals with higher prior AD probabilities and younger ages (Figure‐2A). For example, at a prior AD probability of 70%, the PPV for AD was 92% at ages 50‐55, declining to 87% at ages 85‐90. Tau‐PET NPV was consistently high across ages and prior AD probabilities, effectively ruling out AD (Figure‐2B). Obtaining tau‐PET results after knowing amyloid‐status markedly increased PPV for AD compared to both standalone tau‐PET or amyloid‐PET. The greatest increases in PPV from combining amyloid‐ and tau‐PET, relative to amyloid‐PET alone, occurred with lower pre‐amyloid AD probabilities and older ages (up to a 37% increase; Figure‐3A). Similarly, when tau‐PET was obtained after amyloid‐PET, strong increases in NPV were observed, particularly when prior AD probability was high (up to a 48% increase, Figure‐3D).

**Conclusion:**

Tau‐PET demonstrates high PPV and NPV for clinico‐pathological AD as a standalone marker, with added diagnostic value when amyloid‐status is already known. These findings underscore tau‐PET's value for optimizing the diagnostic process.